# PTGBase: an integrated database to study tandem duplicated genes in plants

**DOI:** 10.1093/database/bav017

**Published:** 2015-03-22

**Authors:** Jingyin Yu, Tao Ke, Sadia Tehrim, Fengming Sun, Boshou Liao, Wei Hua

**Affiliations:** ^1^The Key Laboratory of Biology and Genetic Improvement of Oil Crops, Ministry of Agriculture, Oil Crops Research Institute, Chinese Academy of Agricultural Sciences, Wuhan 430062, China and ^2^Department of Life Science and Technology, Nanyang Normal University, Wolong Road, Nanyang 473061, China

## Abstract

Tandem duplication is a wide-spread phenomenon in plant genomes and plays significant roles in evolution and adaptation to changing environments. Tandem duplicated genes related to certain functions will lead to the expansion of gene families and bring increase of gene dosage in the form of gene cluster arrays. Many tandem duplication events have been studied in plant genomes; yet, there is a surprising shortage of efforts to systematically present the integration of large amounts of information about publicly deposited tandem duplicated gene data across the plant kingdom. To address this shortcoming, we developed the first plant tandem duplicated genes database, PTGBase. It delivers the most comprehensive resource available to date, spanning 39 plant genomes, including model species and newly sequenced species alike. Across these genomes, 54 130 tandem duplicated gene clusters (129 652 genes) are presented in the database. Each tandem array, as well as its member genes, is characterized in complete detail. Tandem duplicated genes in PTGBase can be explored through browsing or searching by identifiers or keywords of functional annotation and sequence similarity. Users can download tandem duplicated gene arrays easily to any scale, up to the complete annotation data set for an entire plant genome. PTGBase will be updated regularly with newly sequenced plant species as they become available.

**Database URL:** http://ocri-genomics.org/PTGBase/.

## Introduction

Angiosperms are an excellent example of a group of plants that provide a sound base for understanding gene duplication (GD) in higher eukaryotes. The history of divergence of the two major classes of angiosperms, i.e. monocots and dicots, goes beyond 125–140 million years ago (MYA) to 170–235 MYA, when the natural tendency of angiosperms towards chromosomal duplication and subsequent gene loss led to much more rapid structural evolution (1–3). All angiosperms underwent polyploidization events, and the fraction of recently duplicated genes is higher in plants than in other eukaryotes (4). These genes originate as a result of at least six different modes of duplication including whole genome, tandem, proximal, DNA-based transposition, retrotransposition and dispersed duplications (5). Among these, tandem duplication refers to the generation of tandem arrays consisting of identical sequences in close genomic proximity and occurs due to unequal chromosomal crossing over (6). In plant genomes, tandem duplication events occur more frequently than other duplication modes and produce greater gene copy number and allelic variation. It is true that the tandem duplication phenomenon affects a small number of genes (∼10% of Arabidopsis or rice genes), but its contribution to the expansion of plant gene families is more significant. In *Arabidopsis* and rice, genes controlling stress tolerance and membrane functions were mostly involved in tandem duplication events (7, 8). Furthermore, tandem GDs have played roles in the evolution of different traits in various plant families like disease resistance in Solanaceae and Brassicaceae (9, 10), signal transduction in legumes (11), glucosinolate biosynthesis diversification in the mustard family (12), and defense response and secondary metabolism like indole alkaloid biosynthesis and tropane, piperidine and pyridine alkaloid biosynthesis in *Brassica oleracea* and *Brassica rapa* (13).

In *Arabidopsis* and *Brassica* species, tandem GD events occurred throughout the evolutionary history, and a whole-genome triplication (WGT) event in *Brassica* did not affect the occurrence of tandem duplication. About 43, 47 and 56% of nucleotide binding site (NBS)-encoding disease resistance (R) genes in *B. oleracea*, *B. rapa* and *Arabidopsis thaliana*, respectively, were generated through tandem GD events; this shows that the rate of tandem duplicated genes is higher in *Arabidopsis thaliana* than in *Brassica* species. Additionally, it was speculated that in Brassicaceae, tandem GD played a more important role in the generation of NBS-encoding R genes than a whole-genome duplication (WGD) event (13, 14). As far as the expression pattern of duplicated genes is concerned, it may follow different outcomes: neo-functionalization (acquire new expression state), subfunctionalization (partitioning of original ancestral function) or pseudo-genization (complete loss of expression) (15–17). In addition, the fate of duplicate retention depends upon certain features like its function, complexity, expression level, network connectivity and dominance of the parental genome (18–25). In angiosperms, tandem duplication-derived evolution can be well studied in Brassicaceae because each of the *Brassica* genomes underwent WGD events and an additional WGT event (specific to the Brassicaceae family); additionally, the close evolutionary relationship between *Brassica* species will facilitate the understanding of the fate of duplicate loss or retention and expression divergence (13, 26).

With the development of sequencing technology, more and more plant genomes were sequenced and released, which provides an opportune chance for researchers to study plant tandem duplicated genes further. Currently, several genomic or transcriptomic data resources for tandem repeats are available online, including STRBase (http://www.cstl.nist.gov/biotech/strbase) (27), TRbase (http://trbase.ex.ac.uk/) (28), TRDB (https://tandem.bu.edu/cgi-bin/trdb/trdb.exe.) (29), TassDB (http://helios.informatik.uni-freiburg.de/TassDB/) (30), VNTRDB (http://vntr.csie.ntu.edu.tw/) (19) and a tandem repeats database for bacterial genomes (http://minisatellites.u-psud.fr) (31). These databases focus on human, bacterial, and some other animal genomes instead of genome sequenced plant species. Tandem repeat DNA sequences, for example SSR and LTR etc., are compiled in these databases except genes performed identical or similar molecular functions. Here, we present PTGBase (freely available at http://ocri-genomics.org/PTGBase/), a database of tandem duplicated genes in assembled pseudomolecules of genome-sequenced plant species, and we demonstrate how this database allows straightforward but flexible searches for tandem duplicated genes or gene clusters in combination with identifiers or keywords of functional annotation (see online supplementary material for Supplementary Table 1). PTGBase is a resource platform through which tandem duplicated genes can be well studied via both intra- and intergenome comparisons to gain insights into their evolutionary history and further explore orthologous and paralogous genes.

## Implementation

**Figure 1.**
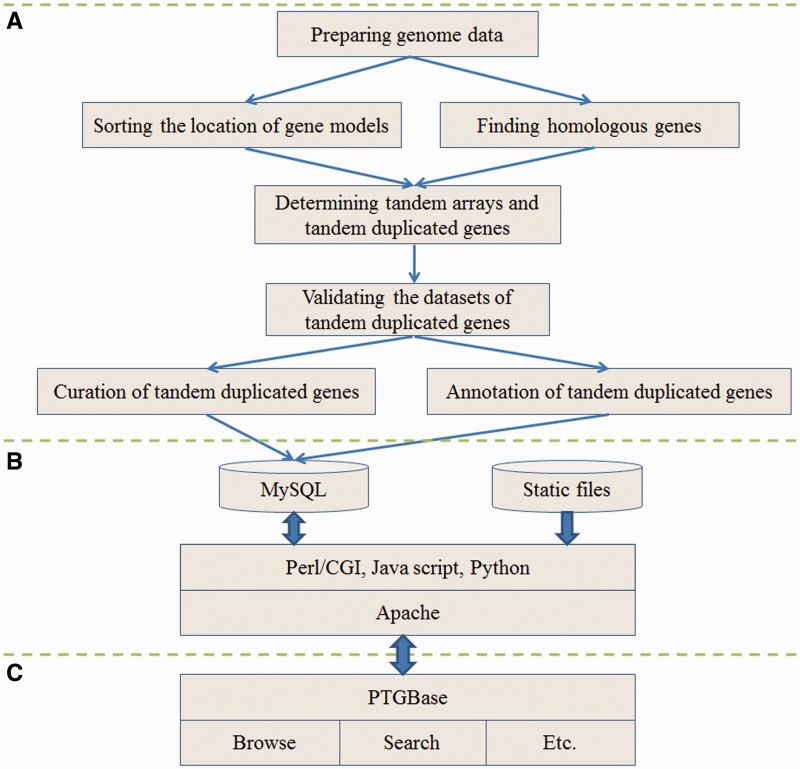
Schematic illustration of the PTGBase sitemap. (**A**) Analysis flowchart to generate the tandem arrays and tandem duplicated genes. (**B**) Diagram of the PTGBase web server. (**C**) Web interface of the PTGBase sitemap.

PTGBase implementation was divided into the following three steps: generate the tandem duplicated genes, set the server configuration and develop a user-friendly interface (Figure 1). Basic datasets of tandem duplicated genes were curated and analyzed by in-house Perl and Python scripts. All basic and annotation information of tandem duplicated genes were stored in the MySQL relational database and static files. PTGBase run on a CentOS operation system with the Apache HTTP server environment and MySQL relational database management system. A user-friendly web interface was developed by Perl and JavaScript programming language. The graphical views of the distribution of tandem duplicated genes on assembled pseudomolecules were developed by Perl GD module from the Comprehensive Perl Archive Network (http://www.cpan.org/) (32). A customized basic local alignment search tool (BLAST), which was downloaded from standard National Center for Biotechnology Information (NCBI) BLAST software package, is implemented to allow users to retrieve homologous genes or regions in corresponding species (33).

## Construction and contents

### Database source

Currently, PTGBase contains 39 plant species with sequenced genomes from important plant families such as Poaceae, Fabaceae, Rosaceae and Brassicaceae. The plant species collected in PTGBase not only include key model plant species for basic scientific research but also important cash crops and food farm crops. Among these 39 plant species, genome data of 28 plant species were downloaded from species-specific databases, including the *Arabidopsis* Information Resource (http://www.arabidopsis.org/) (34) and the *Brassica oleracea* Genome Database (http://ocri-genomics.org/bolbase/) (35). Genome data of the remaining 11 plant species, which were sequenced by the Joint Genome Institute of the US Department of Energy, were downloaded from the Plant Comparative Genomics portal of the Department of Energy's Joint Genome Institute (http://genome.jgi-psf.org/) (36). We extracted four types of files to generate tandem duplicated genes, including sequence files of assembled pseudomolecules, gene model coding sequence files, protein sequence files of gene models and general feature format (GFF) files containing the location of gene models in assembled pseudomolecules (Table 1).

**Table 1.** Statistics of tandem duplicated genes of genome-sequenced plant species in PTGBase

Latin name	Common name	Genome size	Gene number	Number of TD genes	Number of TD clusters	Release version
*Arabidopsis lyrata*	Lyraterockcress	206.7M	32 670	3609	1485	Version 1.0 (Apr 2011)
*Arabidopsis thaliana*	Arabidopsis	125M	35 386	3503	1383	TAIR 9.0 (Jun 2009)
*Aureococcus anophagefferens*	Heterokont algae	57M	11 501	176	84	JGI 1.0 (Sep 2007)
*Brachypodium distachyon*	Purple false brome	260M	31 029	3233	1326	Phytozome v6.0
*Brassica oleracea*	Cabbage	630M	45 758	3443	1514	Version 1.0
*Brassica rapa*	Chinese cabbage	485M	41 173	4501	1918	Version 1.1
*Cajanus cajan*	Pigeonpea	833M	48 680	3837	1736	IIPG v1.0
*Carica papaya*	Papaya	370M	27 950	1937	822	ASGPB v1.0
*Chlamydomonas reinhardtii*	Green algae	130M	17 113	1220	521	Version 4.2
*Chlorella variabilis NC64A*	Microalgae	46M	9 791	451	207	JGI 1.0 (Sep 2010)
*Cicer arietinum*	Chickpea	738M	28 269	1396	610	Version 1.0
*Citrullus lanatus*	Watermelon	425M	23 440	2248	863	Version 1.0
*Citrus sinensis*	Orange	367M	36 450	3253	1261	CITRUS v1.0 (2012)
*Cucumissativus*	Cucumber	350M	26 682	2077	830	Phytozome v6.0
*Fragariavesca*	Strawberry	240M	34 809	3823	1582	GDR v1.0
*Glycine max*	Soybean	1,100M	75 778	5764	2384	v1.1 (Jun 2013)
*Gossypium raimondii*	Cotton	761.4M	40 976	4674	1866	Version 2.1
*Linum usitatissimum*	Flax	373M	43 484	4169	1823	Phytozome v9.1 v1.0
*Lotus japonicus*	Lotus	472M	42 399	1211	543	Release 2.5
*Malus × domestica Borkh.*	Apple	742M	63 541	16 602	6638	GDR v1.0
*Medicago truncatula*	Barrel medic	500M	53 423	3761	1653	Mt3.5 v3 (Jun 2011)
*Musa acuminata*	Banana	523M	36 549	1352	571	CIRAD v1.0
*Oryza sativa L. ssp.jap*onica	Rice	466M	67 393	4544	1931	IRGSP v1.0
*Phaeodactylum tricornutum*	Diatom algae	27.4M	10 402	440	206	JGI 2.0 (May 2007)
*Physcomitrella patens*	Moss	480M	38 354	797	381	Version 1.6 (Jan 2008)
*Populus trichocarpa*	Western poplar	480M	45 033	5224	2084	JGI 2.0 (Feb 2010)
*Prunus mume*	Plum flower	280M	31 390	5059	1985	prunusmumegenome v1.0
*Ricinus communis*	Castor bean	350M	31 221	2613	1075	Release 0.1 (May 2008)
*Selaginella moellendorffii*	Selaginella	212M	22 285	1676	748	Version 1.0 (Dec 2007)
*Sesamum indicum*	Sesame	357M	27 148	2848	1126	Version 1.0
*Setaria italica*	Millet	490M	38 801	4879	2027	Phytozome v9.1
*Solanum lycopersicum*	Tomato	900M	34 727	4173	1640	Version 2.3
*Solanum tuberosum*	Potato	844M	39 031	4504	1839	Version 3.4
*Sorghum bicolor*	Sorghum	730M	29 448	4256	1664	Sbi 1.4 (Dec 2007)
*Thellugiella parvula*	Rockface star-violet	140M	27 132	2316	980	Thellungiella v2.0
*Theobroma cacao*	Cacao	430M	46 143	3867	1604	Release 0.9 (Sep 2010)
*Vitis vinifera*	Grape vine	490M	26 346	3668	1405	Genoscope (Aug 2007)
*Volvox carteri*	Green alga	138M	15 285	1402	595	JGI 1.0 (Jun 2007)
*Zea mays ssp. mays L.*	Maize	2300M	63 293	2837	1220	Release 5a (Nov 2010)

TD, tandem duplicated.

### Finding tandem duplicated genes

In this study, we focused on the tandem duplicated functional genes on the same assembled pseudomolecules excluding one or more unrelated genes within a tandem array, which were generated by tandem duplication or other tandem repeat events. These tandem duplicated functional genes performed identical or similar molecular functions in the process of plant growth, development and adaptation to the environment. In order to get the most accurate datasets of tandem duplicated genes in plants, we designed the following major steps to obtain the tandem duplicated genes from assembled pseudomolecules by procedures consisting of 26 in-house Perl and Python scripts. (i) Finding homologous genes: according to the phylogenetic relationship of all species in different subgroups, at least three species which were belonged to two different layers of phylogenetic tree were recognized as target genomes and one species was regarded as the last common ancestor of other species. The orthoMCL software was used to classify orthologous groups with E-value ≤ 1e-20 and inflation parameter of 1.5, which intended to detect the homologous genes descended for a single gene in the last common ancestor of all species (i.e. the genes descended for a single gene in the last common ancestor of all species under consideration) (33, 37). (ii) Sorting the location of gene models: the GFF file was a key genome sequencing file contained the location of predicted gene models for genome sequenced plant species. Based on GFF files of target genomes, target gene models were sorted by descending order according to the gene location on assembled pseudomolecules. (iii) Determining the tandem duplicated arrays: checking if two or more genes from the same orthologous group are next to each other in target genome. This would allow users to know that the genes are related to each other through duplication without needing to identify the precise taxonomic level at which the GD occurred. (iv) validating the datasets: for the known function duplicated genes, InterPro was employed to validate function of tandem duplicated genes by classifying them into different families and predicting conserved domains and important sites; for the unknown function duplicate genes, a duplicate gene pair was just that the two genes (next to each other in the genome) need to have a sequence coverage percentage ≥80%. (v) Out-putting the standard format datasets: in-house Perl and Python scripts were used to format the output file with cluster names and gene lists. At last, 54 130 tandem arrays (129 652 genes) were generated and curated manually for further analysis (Figure 1A).

### Functional annotation

We generated comprehensive functional annotation of tandem duplicated genes. In PTGBase, all tandem duplicated genes were annotated by performing Blast2GO, a tool for the functional annotation of sequences and the analysis of annotation data (http://www.blast2go.com/), with stringent parameters (38). For each tandem duplicated gene, PTGBase offered complete Gene Ontology (GO) annotation, including GO identifier, term, and corresponding name space. In order to obtain the protein functional classification of tandem duplicated genes, InterPro was employed to provide functional analysis of tandem duplicated genes by classifying them into different families and predicting conserved domains and important sites (39). Every tandem duplicated gene was annotated by the COG database (40). For every tandem duplicated gene, this database supplied InterPro identifiers, functional description and names of member databases in which protein sequences of tandem duplicated genes were classified into families and conserved domain or motif types, as well as identifiers of corresponding member databases in InterPro (Table 2).

**Table 2.** Functional classification of tandem duplicated genes in PTGBase

Latin name	Common name	Numbers of tandem duplicated genes	InterPro		Gene Ontology	
			Gene number	Percentage (%)	Gene number	Percentage (%)
*Arabidopsis lyrata*	Lyraterockcress	3609	3490	96.70	2428	67.28
*Arabidopsis thaliana*	Arabidopsis	3503	3453	98.57	2579	73.62
*Aureococcus anophagefferens*	Heterokont algae	176	160	90.91	121	68.75
*Brachypodium distachyon*	Purple false brome	3233	3149	97.40	2401	74.27
*Brassica oleracea*	Cabbage	3443	3287	95.47	2345	68.11
*Brassica rapa*	Chinese cabbage	4501	4238	94.16	3200	71.10
*Cajanus cajan*	Pigeonpea	3837	3532	92.05	2346	61.14
*Carica papaya*	Papaya	1937	1824	94.17	1353	69.85
*Chlamydomonas reinhardtii*	Green algae	1220	1068	87.54	552	45.25
*Chlorella variabilis NC64A*	Microalgae	451	438	97.12	263	58.31
*Cicer arietinum*	Chickpea	1396	1284	91.98	971	69.56
*Citrullus lanatus*	Watermelon	2248	2168	96.44	1679	74.69
*Citrus sinensis*	Orange	3253	3105	95.45	2385	73.32
*Cucumis sativus*	Cucumber	2077	2024	97.45	1520	73.18
*Fragaria vesca*	Strawberry	3823	3594	94.01	2649	69.29
*Glycine max*	Soybean	5764	5646	97.95	4335	75.21
*Gossypium raimondii*	Cotton	4674	4357	93.22	3235	69.21
*Linum usitatissimum*	Flax	4169	4061	97.41	3012	72.25
*Lotus japonicus*	Lotus	1211	1177	97.19	888	73.33
*Malus × domestica Borkh.*	Apple	16 602	14 085	84.84	10 361	62.41
*Medicago truncatula*	Barrel medic	3,761	3498	93.01	2544	67.64
*Musa acuminata*	Banana	1352	1328	98.22	1082	80.03
*Oryza sativa L. ssp.japonica*	Rice	4544	4374	96.26	3110	68.44
*Phaeodactylum tricornutum*	Diatom algae	440	406	92.27	206	46.82
*Physcomitrella patens*	Moss	797	720	90.34	530	66.50
*Populus trichocarpa*	Western poplar	5224	5100	97.63	3839	73.49
*Prunus mume*	Plum flower	5059	4805	94.98	3551	70.19
*Ricinus communis*	Castor bean	2613	2556	97.82	1908	73.02
*Selaginella moellendorffii*	Selaginella	1676	1483	88.48	954	56.92
*Sesamum indicum*	Sesame	2848	2697	94.70	2073	72.79
*Setaria italica*	Millet	4879	4582	93.91	3255	66.71
*Solanum lycopersicum*	Tomato	4173	3960	94.90	2962	70.98
*Solanum tuberosum*	Potato	4504	4008	88.99	2932	65.10
*Sorghum bicolor*	Sorghum	4256	4169	97.96	3133	73.61
*Thellugiella parvula*	Rockface star-violet	2316	2230	96.29	1686	72.80
*Theobroma cacao*	Cacao	3867	3787	97.93	2858	73.91
*Vitis vinifera*	Grape vine	3668	3596	98.04	2909	79.31
*Volvox carteri*	Green alga	1402	1087	77.53	615	43.87
*Zea mays ssp. mays L.*	Maize	2837	2379	83.86	1647	58.05

## Web interface and usage

### Major modules provided by PTGBase

PTGBase is an integrated plant tandem duplicated genes database that provides not only a comprehensive platform to study plant tandem duplicated genes but also the materials for researchers to further study plant genome evolution. A powerful web-based user interface was designed based on different classifications of major function modules. Each of the major functional modules provided a specific capability for retrieving information about tandem duplicated genes from the database or viewing the tandem duplicated genes in the context of either the phylogenetic or genome sequence comparisons. The two sorting menus show the sum of tandem duplicated gene clusters available from PTGBase by names of plant species. The species names are linked to a list of the associated tandem duplicated gene clusters containing additional information. Depending on the respective focus of data mining, a precise query for identifiers and a fuzzy query for keywords of functional annotations were designed for automated data retrieval of tandem duplicated gene clusters and flexible functional annotations. Moreover, additional functional modules were designed to enrich the content of PTGBase and supplied a comprehensive resource platform of tandem duplicated genes for the community.

### Browse module to show overall view of tandem duplicated genes and clusters

**Figure 2.**
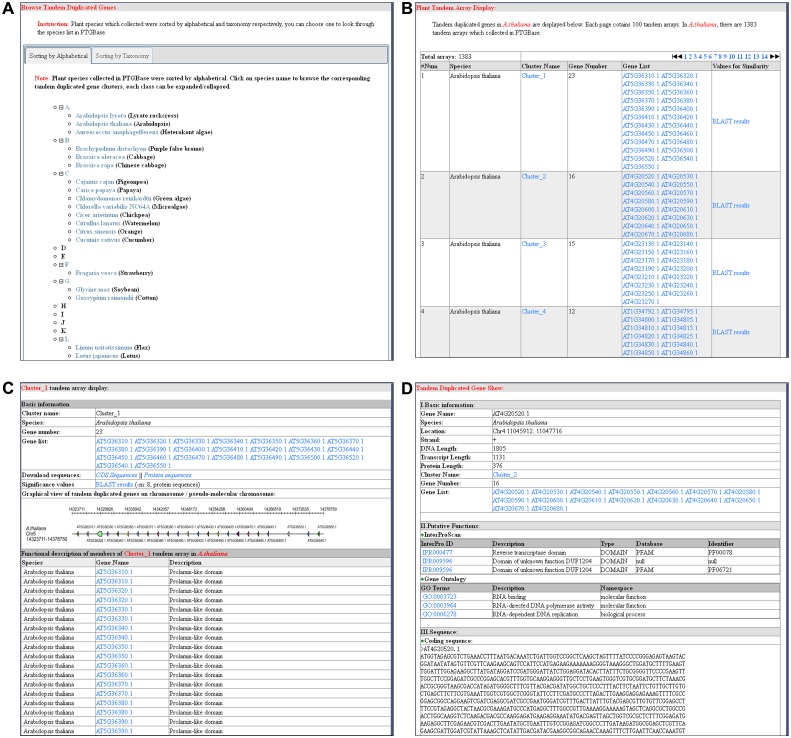
Major browsing function modules of PTGBase. (**A**) Overview of browsing functions for tandem arrays in PTGBase. (**B**) Browsing the tandem duplicated genes by tandem array in special plants. (**C**) Detailed annotation of a tandem duplicated gene cluster. (**D**) Detailed annotation of tandem duplicated genes in PTGBase.

Multilayer browse modules were developed to display a comprehensive resource of tandem duplicated genes compiled in PTGBase (Figure 2). There are 39 plant species deposited in PTGBase; standardizing the order of these plant species will bring more convenience to select the data for species of interest. The browse module offers two major navigation tabs to show plant tandem duplicated gene clusters: (i) alphabetical sorting and (ii) sorting by taxonomy (Figure 2A). In the alphabetical sorting, all species are sorted alphabetically, and every class can be expanded or collapsed by clicking the corresponding icons. Following the evolutionary relationship deposited in corresponding genome papers and the NCBI taxonomy database, we constructed the phylogenetic tree among plant species in PTGBase. In the sorting by taxonomy tab, a phylogenetic tree is provided to show plant species that supply a clear evolutionary pedigree for users to further study the evolutionary history of tandem duplicated genes. In the two tabs, users can select a species of interest and click the species name to retrieve the tandem duplicated gene clusters in the selected species. Tandem duplicated gene clusters are shown with the following five pieces of information: species to which the clusters belong, cluster name, number of genes in the clusters, gene list in clusters, and significance values for sequence similarities (Figure 2B). Clicking the hyperlink of the cluster name allows users to obtain the information of this whole tandem duplicated gene cluster which includes the number of genes in the cluster, coding and protein sequences of duplicated genes, significance values for sequence comparison, distribution of the duplicated genes on assembled pseudomolecules, and functional features of duplicated genes (Figure 2C). The distribution of duplicated genes on pseudomolecules provides a valuable hint about the formation of the duplicated genes on assembled pseudomolecules and improves the understanding of the tandem duplicated genes. The summary of functional features of duplicated genes indicates the functional types of duplicated genes that are clustered together on assembled pseudomolecules. Clicking the hyperlink of the duplicated gene name displays basic information, putative function, and sequence information in detail (Figure 2D). Multi-level browse functional modules will allow more opportunities for users to identify useful information and understand tandem duplicated genes clearly.

### Search module for identifiers or keywords in the database

Searching the function module of identifiers related to tandem duplicated genes or keywords of functional annotation was developed by Perl and JavaScript scripts which supplied a visual and powerful searching platform. A search navigation is available at the top of the searching function module, providing a quick and clear means for searching specific objects by identifiers or keywords in this database. According to different entry points to search, three parts were deposited in PTGBase which contained searching by identifiers or names of tandem duplicated genes, searching by identifiers of functional annotations and searching by keywords of functional annotations. In the section to search by identifiers or names of tandem duplicated genes, users can retrieve valuable information about tandem duplicated genes by inputting a gene ID, a whole tandem array of tandem duplicated genes by supplying the name of a gene and cluster and a tandem array list by supplying gene numbers and species name. Users can also retrieve basic information about tandem duplicated genes compiled in PTGBase. In the section to search by identifiers of a functional annotation, users can supply a GO ID or InterPro accession number to obtain tandem duplicated genes with those annotations among species that can be used to understand certain functional types of clustered genes on assembled pseudomolecules. Moreover, the search module also allows users to use keywords of functional annotations to search tandem duplicated genes among species. A powerful fuzzy search function was developed that permits users using simple keyword of a functional annotation to traverse the whole annotation database to obtain all duplicated genes containing the simple keyword in their functional description.

### Sequence similarity search by nucleic acid or amino acid sequence(s)

Customized WWWBLAST modules were designed for users to implement online sequence comparison conveniently in PTGBase (33). The query is a nucleic acid or an amino acid sequence. By uploading a sequence file or pasting a sequence directly, users can find homologous duplicated genes or syntenic regions from compiled genome datasets by selecting an appropriate BLAST program and designated plant species. Thus, by implementing a sequence similarity search, users can obtain not only the putative annotation of the query sequence but also the location of the query sequence on assembled pseudomolecules by homology sequence comparison. For BLAST hits, hyperlinks to the annotation pages in PTGBase and cross-links to annotation pages in the species-specific database have been added in this database for users to get more annotations of query sequence.

### Download tandem duplicated genes data and contribution to PTGBase

PTGBase supplies a convenient download module for users to retrieve useful information about tandem duplicated genes. First, users can download a compressed file containing tandem duplicated gene clusters and coding or protein sequences of tandem duplicated genes by selecting a target plant species in the box and clicking the ‘download’ button. Second, genome data of genome-sequenced plant species collected in PTGBase can be downloaded freely, and the data policy of the released genome should be obeyed. The downloadable genome data contain coding and protein sequences and a GFF file containing the location of gene models on assembled pseudomolecules. If users want other files of genome data for species of interest, they can access the hyperlinks of the species-specific expert database or JGI, which is supplied by our database, to obtain complete genome sequencing data for the plant species of interest.

In order to supply an excellent data resource of tandem duplicated genes in plants for the community, PTGBase asked users to submit the tandem duplicated genes to this database and enriched the contents of tandem duplicated genes in PTGBase. The procedures to generate the tandem duplicated genes should follow the pipeline of PTGBase. Moreover, we can help users obtain tandem duplicated genes for their species of interest. After curation, the newly available data of tandem duplicated genes will be included in PTGBase.

## Discussion

PTGBase represents an exhaustive collection of plant tandem duplicated genes that were collected and compiled from several public databases and additional private resources. It will present an unprecedented opportunity to study gene family expansion of specific traits or phenotypes and plant intra- and intergenome evolution. When a class of genes that performs a specific function experienced tandem duplication or other tandem repeat events after the formation of a species, it will increase the gene dosage, which enhances the gene function and results in either beneficial or detrimental effects on plant growth, development or adaptation to the environment (41, 42). For example, NBS-encoding genes play an important role in resistance to diseases and are greatly influenced by tandem duplication. In a recent study, Yu *et al.* (2014) systematically reported that NBS-encoding genes in *Brassica* species experienced species-specific gene family amplification by tandem duplication after the divergence of *B. rapa* and *B. oleracea*. LRR-RLK genes is another type of disease resistance genes (R genes) and have a critical role in defense response. Argout *et al.* (43) reported that the *Theobroma cacao* genome contains at least 253 LRR-RLK genes orthologous to Arabidopsis LRR-RLK genes. According to the analysis of tandem duplicated genes for *T. cacao* genome in PTGBase, 46 LRR-RLK genes were generated by tandem duplication event, representing approximately 18.2% of total LRR-RLK genes in *T. cacao* genome. For R genes, the tandem duplication event will increase the gene dosage and the increased gene dosage might have some advantages to plant pathogen defense (14).

The emergence of tandem duplicated genes has given rise to great challenges for studying orthologous genes among species in the context of plant evolution. After the divergence of plant species from ancestral species, plant species have experienced tandem duplication events and formed species-specific tandem repeat or duplicated genes. The most straightforward way to detect orthologous genes of tandem arrays between different species is to use a sequence similarity search to classify orthologous genes among tandem arrays of different species. The expression patterns of repeat or duplicated genes reveal different outcomes: neo-functionalization, subfunctionalization and pseudogenization (15–17). Yu *et al.* (14) examined the expression profile of NBS-encoding genes of a tandem array and explored the hypothesis that the expression profiles of different NBS-coding members are separated into different groups that are indicative of functional divergence, but the members of an NBS-encoding tandem array performed the same function with the same gene expression pattern and shared nearly identical sequence similarity. Therefore, using a sequence similarity search was the best way to explore the orthologous genes of tandem arrays among different species until now.

## Conclusions and perspectives

PTGBase is the first plant tandem duplicated genes database that embraces a wide spectrum of genome resources for genome-sequenced plant species. It not only focuses on functional genomics for each plant species but also is dedicated to comparative genomics in the context of plant phylogenetic analysis spanning a wide range of plant genomes. PTGBase provides effective data mining tools and efficient use of tandem duplicated gene information for users to retrieve useful data easily. The database will be continuously improved by updating tandem duplicated gene collections and newly detected tandem duplicated genes from available plant genomes within the framework of PTGBase. Future efforts will also develop better approaches to classify plant genes correlated with tandem duplicated events, as well as refine the structure of this database. We aim to develop and maintain a comprehensive plant tandem duplicated genes database to improve our knowledge of functional genomics, comparative genomics, and evolutionary biology by providing systematic data resources and integrative analytical frameworks and views.

## Supplementary Data

Supplementary data are available at *Database* Online.

Supplementary Data
