# Supplementary material for: PTGBase: an integrated database to study tandem duplicated genes in plants
Source: Database (Oxford). 2015 Mar 22;2015:bav017. doi: 10.1093/database/bav017 (PMC4369376; doi:10.1093/database/bav017)
Supplement: Supplementary Data [file supp_2015_bav017_index.html]

PTGBase: an integrated database to study tandem duplicated genes in plants — Supplementary Data 

# PTGBase: an integrated database to study tandem duplicated genes in plants

## Supplementary Data

files

**Files in this Data Supplement:**

- Supplementary Data - xlsx file
